# IMMUNIZATION AS A HEALTHY AGING STRATEGY

**DOI:** 10.1093/geroni/igz038.773

**Published:** 2019-11-08

**Authors:** Eduardo De Gomensoro

**Affiliations:** GSK Vaccines, Rockville, Maryland, United States

## Abstract

The burden of infectious disease is heavier at the extremes of life: the youngest and the oldest typically have the highest incidence of infectious diseases along with morbidity and mortality. Demographic studies show by the next decade, adults over 65 will outnumber children under 5 years of age. Unfortunately, vaccination coverage in older adults even where permissive recommendations exist is universally lower than in infants or children. Key reasons are a lack of knowledge and understanding of the benefits of vaccination, and inconsistent recommendations by providers. Recently, the concept of ‘healthy aging’ – regular vaccination and lifestyle modification including exercise and diet – has been proposed to go beyond disease prevention and address quality of life issues such as the ability to remain in work, and to live independently. Public and healthcare provider education to ascertain the value of older adult’s immunization are critical for the fulfilment of this agenda.

